# Serological Detection of Marine Origin Brucella Exposure in Two Alaska Beluga Stocks

**DOI:** 10.3390/ani12151932

**Published:** 2022-07-29

**Authors:** Laura A. Thompson, Caroline E. C. Goertz, Lori T. Quakenbush, Kathy Burek Huntington, Robert S. Suydam, Raphaela Stimmelmayr, Tracy A. Romano

**Affiliations:** 1 Mystic Aquarium, Division of Sea Research Inc., Mystic, CT 06355, USA; tromano@mysticaquarium.org; 2 Alaska SeaLife Center, Seward, AK 99664, USA; carrieg@alaskasealife.org; 3 Alaska Department of Fish and Game, Fairbanks, AK 99701, USA; lori.quakenbush@alaska.gov; 4 Alaska Veterinary Pathology Service, Eagle River, AK 99577, USA; avps.kbh@gmail.com; 5 North Slope Borough Department of Wildlife Management, Utqiagvik, AK 99723, USA; robert.suydam@north-slope.org (R.S.S.); raphaela.stimmelmayr@north-slope.org (R.S.); 6 Institute of Arctic Biology, University of Alaska Fairbanks, Utqiagvik, AK 99775, USA

**Keywords:** *Brucella*, beluga, serology, disease, rtPCR, bacterial culture

## Abstract

**Simple Summary:**

Brucellosis, the disease caused by *Brucella* bacteria, is of emerging concern in marine-mammal populations worldwide due to its potential link to reproductive failure, yet is less well-studied than in terrestrial animals, such as cattle. To understand *Brucella* exposure and disease in two populations of beluga, in Bristol Bay and the eastern Chukchi Sea, Alaska, USA, this study screened animals for the presence of antibodies against the bacterium (serology), as well as tested for the direct presence of bacterial DNA or bacterial growth from tissue samples. More than half of all animals tested, from both populations, were positive for the presence of antibodies, providing evidence of exposure to *Brucella*. Few animals, however, were positive for the direct detection of *Brucella* DNA and none resulted in successful bacterial growth, suggesting a lack of active clinical disease. The high rate of exposure in these populations supports the need for long-term monitoring of beluga populations, particular those that are threatened or endangered, such as the Cook Inlet belugas.

**Abstract:**

Among emerging threats to the Arctic is the introduction, spread, or resurgence of disease. Marine brucellosis is an emerging disease concern among free-ranging cetaceans and is less well-studied than terrestrial forms. To investigate marine-origin *Brucella* sp. exposure in two beluga stocks in Alaska, USA, this study used serological status as well as real-time polymerase chain reaction (rtPCR) and bacterial culture. In total, 55 live-captured–released belugas were tested for *Brucella* exposure in Bristol Bay (2008–2016) and 112 (8 live-captured; 104 subsistence-harvested) whales were tested in the eastern Chukchi Sea (2007–2017). In total, 73% percent of Bristol Bay live captures, 50% of Chukchi Sea live captures, and 66% of Chukchi Sea harvested belugas were positive on serology. Only 10 of 69 seropositive belugas were rtPCR positive in at least one tissue. Only one seropositive animal was PCR positive in both the spleen and mesenteric lymph node. All animals tested were culture negative. The high prevalence of seropositivity detected suggests widespread exposure in both stocks, however, the low level of rtPCR and culture positive results suggests clinical brucellosis was not prevalent in the belugas surveyed. Continued detection of *Brucella* exposure supports the need for long-term monitoring of these and other beluga populations.

## 1. Introduction

Beluga whales (*Delphinapterus leucas*) are a mid-size odontocete with arctic and sub-arctic distribution. There are 22 recognized stocks of belugas, 6 of which are of some national concern [1]. Environmental changes due to a rapidly warming climate allow increased accessibility for shipping, resource exploration, and exploitation, as well as tourism that creates disturbance-related challenges for belugas [2,3,4]. In addition to these threats is the threat of emerging and re-remerging disease, which can occur when environmental changes allow the spread of new diseases or increases in existing diseases. Such exposure can be particularly devastating for immunologically naive populations. The role of infectious diseases among beluga stocks is less well-understood, however, climate change is predicted to increase infectious disease outbreaks and extent among marine-mammal populations [for review see 4]. Among the various viral and bacterial pathogens known to affect cetaceans, marine *Brucella*, in particular *B. ceti*, is an emerging disease concern among free-ranging cetaceans worldwide and in North America [5].

Marine *Brucella* sp. were first identified in marine mammals in 1994, reported in a bottlenose dolphin (*Tursiops truncates*) [6], as well as harbor seals (*Phoca vitulina*), harbor porpoises (*Phocoena phocoena*), and common dolphins (*Delphinus delphis*) [7]. Since then, exposure to *Brucella* has been reported in a variety of marine-mammal species including Arctic and Antarctic populations [8]. The genetic variability of marine *Brucella* is greater than their terrestrial counterparts and multiple strains of marine *Brucella* have been reported, with some host preference for cetaceans and pinnipeds [8,9,10], however this preference is not absolute [11].

Marine *Brucella* sp. infection in cetaceans shows a broad and variable disease presentation and may occur without clinical indication of disease, making it difficult to interpret the significance of exposure. Marine brucellosis has been associated with lesions in the brain, cardiovascular system, lungs, bones, skin, and reproductive organs [5,12,13,14,15,16]. Reproductive failure is of particular concern for species with inherently low reproductive potential and for threatened and endangered marine-mammal populations. *Brucella* sp. have been identified in the reproductive organs of both male and female cetaceans, and have been associated with abortions and stillbirths in bottlenose dolphins [6,15,17,18] and dwarf sperm whales (*Kogia sima*) [19]. *Brucella* sp. have also been implicated in placental disease in northern fur seals (*Callorhinus ursinus*) [20].

Most of the terrestrial *Brucella* strains are known to be zoonotic; however, the zoonotic potential of marine *Brucella* sp. remains ill-defined, with only a few cases of marine *Brucella* infection with *B. ceti* sequence type 27 documented in humans [15,21]. ST27 has been identified in bottlenose dolphins, common dolphins, sperm whales (*Physeter microcephalus*), dwarf sperm whales and California sea lions (*Zalophus californianus*) [5,11,19]. In Alaska, *B. pinnipedialis* is the only strain that has been identified in belugas [11]. However, *B. pinnipedialis* infection in cetaceans has rarely been associated with pathology [22,23]. Furthermore, *Brucella* ST 27 has been identified in a single Steller sea lion (*Eumetopias jubatus*) in Alaska’s waters [24], highlighting the need for continued monitoring.

The gold standard for *Brucella* detection is bacterial culture, which can be difficult due to the need for fresh samples that require special handling and culture requirements, such as CO_2_ between strains [25] and a laboratory with high-level biosecurity controls. Serological samples are easier to collect in the field; however, assays can only be used to detect the presence of antibodies that indicate exposure to *Brucella* at some point in time and do not provide evidence of the occurrence of disease within a population. Serological assays carried out for belugas in the Sea of Okhotsk reported low exposure (<20% seropositive) [26,27]. One location in the Canadian Arctic showed a higher rate of 35.7% (Nunavik) [28], although other locations within the Canadian Arctic were lower.

These previous studies relied on tests developed for terrestrial *Brucella* sp. such as *B*. *melletensis* and *abortus,* which have been shown to have variable results in detecting exposure to marine origin *Brucella* [28,29]. This study used a competitive enzyme linked immunosorbent assay (ELISA), developed with a marine *Brucella* isolate, to investigate the prevalence of marine *Brucella* sp. exposure in two beluga stocks in Alaska, USA (see methods). To the best of our knowledge, this is the first major survey of *Brucella* exposure in belugas from Bristol Bay (BB) and the eastern Chukchi Sea (CS), Alaska.

## 2. Materials and Methods

### 2.1. Animal Sampling

#### 2.1.1. Live-Capture—Release (Bristol Bay and Eastern Chukchi Sea)

Blood samples were obtained from belugas during live-capture health assessments in 2008, 2012, 2013, 2014, and 2016 in Bristol Bay and each year during 2007–2017 near Point Lay, Alaska, in the eastern Chukchi Sea. In Bristol Bay, samples were collected in the spring (May) during 2008 and 2016, and in the fall (August/September) during 2008, 2012, 2013, and 2014. In the Chukchi Sea, samples were collected during the summer (June–July). Small boats were used to guide belugas into shallow water, and nets were used for capture of individual whales. Animals were quickly removed from nets, and manually restrained for tagging and health assessments, including blood sampling [30,31,32]. Blood was drawn for serology and hematology from the superficial fluke veins.

#### 2.1.2. Subsistence Harvested (Eastern Chukchi Sea)

Blood samples were obtained from subsistence-harvested belugas from the peduncular vein and/or intracardially. Spleen (SPL) and mesenteric lymph node (MLN) samples were collected from a subset of harvested belugas for real-time PCR (n = 50) and bacterial culture (n = 22). Tissue samples were flash-frozen. All samples were frozen and shipped in a liquid nitrogen (LN) dry shipper to Mystic Aquarium, Connecticut, for analyses.

### 2.2. Serology

Blood was collected in serum separator vacutainer tubes (SST) and allowed to clot for at least 1 h, or until return to the laboratory from the field-sampling sites. Tubes were centrifuged for 10 min at 2000× *g*, and serum was frozen in 1 mL aliquots for transfer to Mystic Aquarium. Serum was stored at −80 °C until assayed. Exposure to *Brucella* sp., was investigated through use of a competitive ELISA (cELISA) developed at Mystic Aquarium [33]. This assay was developed with whole antigen, identified as *B. pinnipedialis*, isolated from a harbor seal, and has reported high sensitivity and specificity as compared with terrestrial assays. Briefly, serum samples were diluted 1:10 and incubated with the *Brucella* antigen. A biotinylated goat anti-*Brucella* antibody was then added, which competes with native antibody in the sample. Later steps in the assay build a colorimetric signal from the biotin. Final plate development was read at 450 nm on an EL800microplate reader (2008–2013) or an Epoch plate reader (2014–2017; Biotek Instruments, Winooski, VT, USA). The percent inhibition (%inhibition) was calculated based on a ratio of positive (positive goat serum), negative (negative goat serum), and no serum (assay incubation buffer) controls. Positive results are indicated by a %inhibition above 30. Animals that fell in the suspect range, %inhibition between 25 and 30, were treated separately from the positive group. Negative results are indicated by a %inhibition below 25.

### 2.3. Real-Time PCR

MLN and SPL tissue samples were cut into 1 cm^3^ pieces and flash-frozen. A multiplex real time PCR (rtPCR) assay, previously developed at Mystic Aquarium, was used to detect the presence of *Brucella* sp. bacterial DNA in these tissues [34]. This assay utilizes the *bcsp31* gene for the detection of *Brucella* sp. as well as an internal salmon control and DNA quality control. DNA was extracted from tissues using the DNeasy Blood and Tissue Kit from Qiagen Inc. (Valencia, CA, USA). Proteinase K was used during DNA extraction to digest and remove protein or other potential contaminants. Amplification cycles were initiated at 95 °C for 15 min, followed by denaturation and annealing phases for 30 and 60 s, respectively. Denaturation and annealing occurred over 45 repeated cycles for a total running time of approximately 2 h 9 min. Quality control results were determined based on amplification of the internal salmon and DNA quality controls. The fluorescence threshold for *bcsp31* gene was set at 50,000, and samples with a sigmoidal curve rising above the threshold after cycle 29 were determined to be positive (based on [34]).

### 2.4. Tissue Culture

Frozen MLN and SPL were also used for bacterial culture. Approximately 5 g subsections of tissue were thawed, flame-sterilized, and homogenized in 5 mL of sterile phosphate-buffered saline. Homogenates were streaked onto tryptic soy agar plates [35] and incubated at 37 °C and 10% CO_2_ for 2 weeks. Culture plates were checked approximately every 3 days throughout incubation to record growth. Suspected growth included circular colonies with smooth margins and slight opalescence.

### 2.5. Hematology

Whole blood was collected in EDTA vacutainers for analysis using a VetScan HM2 Hematology Analyzer (Abaxis Zoetis, Union City, CA, USA). Parameters considered in this study include WBC, RBC, HGB, HCT, MCV, MCH, Plat, PCT, MPV, PDWc, and Fibrinogen [31].

### 2.6. Statistical Analyses

Results of serology are reported using descriptive statistics for each of the two beluga populations included in this study. For the purposes of this study, suspect results (between 25 and 30% inhibition) were not included as positives. The difference in occurrence of serological positives between populations was explored using a likelihood-ratio chi-squared test, as were sex and age effects. For these tests, occurrence data were entered in three categories: negative (%inhibition < 25), suspect (%inhibition > 25 and <30), and positive (%inhibition > 30). Sex differences in seropositivity were investigated for Bristol Bay and eastern Chukchi Sea populations individually, as well as for the combined data. To investigate the effect of age, belugas were categorized in two age groups based on length, adult (>310 cm) and immature (<310 cm). Estimates of sexual maturity for belugas vary, and this length was chosen to approximate ages of 7 to 9 years old [30]. For Bristol Bay live-capture–released animals, the relationship between hematology in serologically negative, positive and suspect was investigated using independent sample Kruskal–Wallis tests. For all statistics, alpha was set at 0.05.

## 3. Results

### 3.1. Bristol Bay

A total of 55 belugas sampled in Bristol Bay over the course of the study were tested for *Brucella*. Positive serology was detected in 73% of animals (40/55), including 100% of animals sampled during 2008 and 2012 (Table 1). In total, 13 of 24 females had high serum progesterone (≥10 ng/mL) and were considered pregnant [32], and 46% (6/13) of these animals were seropositive for *Brucella*. In the spring, 16 of 19 belugas (84%) were seropositive regardless of sex, and in the fall 26 of 36 belugas (72%) were seropositive (x^2^_(2)_ = 204.71, *p* < 0.001). Hematology parameters did not differ significantly between seropositive, negative, and suspect animals.

### 3.2. Eastern Chukchi Sea

A total of 8 live-capture–released and 104 subsistence-harvested belugas from the eastern Chukchi Sea population were tested, and 65% were seropositive, including 50% of those that were live-captured (4/8) and 66% of those that were harvested (69/104) (Table 2). All harvested belugas during 2007, 2011, and 2012 were positive. Tissue samples from 50 animals were tested using rtPCR, and 10 were positive. Two seronegative animals were PCR positive in SPL tissue. Seven animals that were seropositive were also PCR positive; six were PCR positive in only one tissue (SPL positive n = 4; MLN positive n = 2), while only one seropositive animal was PCR positive in both the spleen and MLN. An additional animal was PCR positive in both tissues, however, serology results were unavailable. No cultures were positive.

### 3.3. Population Comparison

No significant difference was detected in the occurrence of positive exposures between populations (*p* > 0.05).

### 3.4. Effect of Age and Sex

There was no difference in the occurrence of seropositives between sexes, either for the total or within each population (*p* > 0.05). Overall, there was no significant effect of age on seropositivity, however, within Bristol Bay alone there was a significant likelihood ratio (x^2^_(2)_ = 202.74, *p* < 0.001) based on age, and animals classified as immature were either positive or suspect.

## 4. Discussion

Our results demonstrate higher rates of seropositivity (Bristol Bay, 73%; Chukchi Sea, 65%) as compared to studies carried out in Sakhalin Bay and the Sea of Okhotsk, Russia, the Canadian Arctic, and Nunavik, Hudson Bay, Baffin Island, and the Mackenzie Delta. This high occurrence of seropositivity may be in part explained by differences in methodological approaches. The current study utilized a competitive ELISA with high specificity and sensitivity for marine *Brucella* [29], while previous studies utilized reagents for terrestrial *Brucella* sp. While *Brucella* sp. demonstrate high genetic relatedness, marine strains have been classified as a distinct cluster from terrestrial strains (Figure 2 in [36]). Variability among assays has been demonstrated including the potential for terrestrial assays to return false negatives in the case of marine *Brucella* exposure [37].

No significant difference in *Brucella* exposure was detected between males and females, and this is similar to findings in other marine-mammal species [38]. Both beluga sampling efforts in this study were limited to larger (i.e., older) animals. Live captures in Bristol Bay for example, had a minimal length requirement of 250 cm for sampling [32], corresponding to an age of approximately five or six years old [30], and subsistence hunters tend to harvest larger white belugas (i.e., adults); thus, age comparisons were limited for this study. Nonetheless, a few animals included in this study from both populations were immature with shorter reported lengths and were documented as gray to dark gray in color. Our finding that immature belugas had an increased likelihood of exposure is similar to other findings for belugas [28] and hooded seals [38]. Vertical transmission of Brucella from mother to young, and the presence of *B. ceti* in milk, has been reported in some marine mammals [15,39] and may play a role in age-related serological status. This was likely not a factor in the hooded seal study, however, as the authors note that *B. pinnipedialis* has never been isolated from adults of this species [38]. Instead, Nymo et al. [38] postulate that an age effect may be due to transmission of *Brucella* sp. by prey (fish or fish parasites), which are first encountered during weaning, with subsequent clearance of infection with age. Given the limited age distribution in our study, we cannot address this aspect, but suggest that future surveys should consider ways to include younger animals or test milk samples where possible, for example, from subsistence-harvested whales.

Serological results suggest a majority of the belugas in this study were exposed to marine *Brucella* sp. It is interesting that a significantly higher occurrence of seropositivity was detected in the spring as compared with the fall in Bristol Bay belugas. Body condition, as indicated by blubber stores, may be poorer in the spring [40], leaving belugas more susceptible to the spread of disease. In our study, however, the body-condition index of length/girth was not significantly different between seasons. We did, however, find that immature whales (smaller length) in this study had a higher likelihood of seropositivity, and smaller animals made up a larger portion of those sampled during the spring as compared with the fall. However, there were fewer animals sampled in the spring compared to the fall (19 vs. 36), and different seasons were sampled in different years, thus, this may be an artifact of uneven group sizes or temporal trends. Increased detection of *Brucella* sp. during the spring has been reported for both terrestrial mammals and dolphins, and is thought to be related to breeding and calving seasons, possibly due to spread through mating activity or the production of milk [41]. Beluga calves are born in the spring and summer, thus, our findings are consistent with these previous studies.

*Brucella* infection in cetaceans is diagnosed by histology, immunohistochemistry, bacterial culture, PCR, and serology [5,42]. Serology, albeit a useful diagnostic surveillance tool, is insufficient by itself to determine active infection. It is unknown how long antibodies to marine Brucella may last in marine mammals; however, studies with terrestrial strains indicate antibodies may circulate for several years after exposure [25]. Direct identification of *Brucella* sp. is necessary to determine infection [43], which currently can only be achieved through isolation of bacterial DNA and molecular identification (PCR) or bacterial culture of tissue samples.

Tissue culture and rtPCR were not conducted for live-capture–released belugas from either study population, however, rtPCR was conducted for MLN and SPL tissues for subsistence-harvested eastern Chukchi Sea belugas. Despite a high occurrence of seropositivity, there were relatively few positive rtPCR results. Concerns over sample degradation, due to the time after harvest that tissues could be collected, were addressed through quality controls built into the rtPCR assay used here. During development of this assay, no loss of detection of *Brucella* bacterial DNA was identified after 16 days of tissue degradation [34]. Two seronegative animals tested positive for *Brucella* amplification in the spleen on PCR. These two individuals may also represent an early phase of infection, where the antibody may not yet be detectable.

Lymph nodes and spleen are among the tissues that have been identified as ideal for *Brucella* sp. detection, as the bacteria infect and replicate in the macrophage and endothelial cells associated with the reticuloendothelial system [12]. Additional tissue types may be of interest in screening for *Brucella* sp., particularly if there is clinical evidence of disease [25]. Reproductive tissue, for example, may of interest in cases of fetal abortion. For the purposes of this study, testing of additional tissue types was not targeted due to field logistics during sampling, and a lack of evidence of disease or reproductive failure in the study population. Nonetheless, the spleen and LN are highly reliable for the recovery of *Brucella* sp. and likely play a role in maintaining and transporting infection throughout the body [12], thus making them good candidates for this study.

No animals were culture positive, suggesting that false negatives were not being obtained by rtPCR. This likely reflects differences in the sensitivities of different diagnostic approaches. Godfroid et al. [25] indicates that PCR approaches may have better sensitivities compared with culture. Together, these data support the conclusion that while *Brucella* sp. exposure is high, the strain in question may be nonpathogenic, or belugas may be able to clear infections. Increased *Brucella suis* detection has been reported in declining populations of caribou in Alaska [44]. While there may be some environmental exposure for marine-mammal populations that frequent shallow coastal waters that border caribou habitat, to the best of our knowledge the detection of terrestrial *Brucella* sp. has not been reported in marine mammals. In fact, marine *Brucella* sp. are distinct from terrestrial strains, and there is currently no evidence of transformation between strain types [45,46].

The combination of high seropositivity with low PCR positivity in this study may suggest that *Brucella* infection is endemic and likely self-limiting in both populations [43]. High abundance and stable-population trends for both these stocks [47] provide support for our conclusion. A multiyear survey of Alaskan pinnipeds reported seropositivity in 28 of 45 harbor seals sampled in Bristol Bay, but did not report any results suggesting that *Brucella* had a major impact on population dynamics [48]. In addition, the lack of significant hematological changes between seropositive and seronegative groups in our study also suggests the absence of an active response to infection at the time of sampling.

## 5. Conclusions

In summary, a high prevalence of belugas seropositive for antibodies against marine *Brucella* sp. was detected in two populations inhabiting Alaska’s waters; however, few animals were positive on PCR and none on culture. Failure to culture the organism limited our ability to further characterize the detected *Brucella* strain. The continued detection of *Brucella* exposure over time in these two Alaskan beluga stocks supports the importance of including *Brucella* surveys in long-term monitoring efforts and further efforts to characterize the circulating marine *Brucella* sp. strains found in belugas. In addition, the Bristol Bay population of belugas is often used as a proxy for the endangered Cook Inlet belugas, due to its geographical and genetic similarities [49]. Thus, understanding marine *Brucella* disease dynamics in Bristol Bay can be useful for assessing health threats to the Cook Inlet belugas. Lastly, given the nutritional and cultural importance of belugas for circumpolar indigenous communities, monitoring for *Brucella* sp. in marine mammals that are harvested for subsistence will remain important from a ONE-health perspective [50].

## Figures and Tables

**Table 1:** Summary of *Brucella* serology results for live-capture belugas sampled in Bristol Bay, AK.

	2008	2012	2013	2014	2016	Total
Negative	0	0	2	8	0	** 10 **
Positive	18	9	5	2	6	**40**
Suspect	0	0	2	0	3	**5**
**% Positive**	**100**	**100**	**55.56**	**20**	**66.67**	**72.73**

**Table 2:** Summary of Brucella serology results for eastern Chukchi Sea belugas.

		2007	2008	2009	2010	2011	2012	2013	2017	Total
Harvested	Negative	0	5	3	7	0	0	3	7	**25**
	Positive	12	12	7	10	5	6	5	10	**67**
	Suspect	0	3	1	2	0	0	3	1	**10**
	**% Positive**	**100**	**60**	**64.64**	**52.63**	**100**	**100**	**45.45**	**55.56**	**65.68**
Live Capture	Negative	1	0	0	1	0	0	0	1	**2**
	Positive	1	0	0	1	0	0	0	2	**4**
	Suspect	1	0	0	0	0	0	0	0	**1**
	**% Positive**	**33.33**	**0**	**0**	**50**	**0**	**0**	**0**	**66.67**	**50**

## Data Availability

Not applicable.
